# LipL41, a Hemin Binding Protein from *Leptospira santarosai* serovar Shermani

**DOI:** 10.1371/journal.pone.0083246

**Published:** 2013-12-12

**Authors:** Ming-Hsing Lin, Yuan-Chih Chang, Chwan-Deng Hsiao, Shih-Hsun Huang, Min-Shi Wang, Yi-Ching Ko, Chih-Wei Yang, Yuh-Ju Sun

**Affiliations:** 1 Institute of Bioinformatics and Structural Biology, National Tsing Hua University, Hsinchu, Taiwan; 2 Institute of Cellular and Organismic Biology, Academia Sinica, Taipei, Taiwan; 3 Institute of Molecular Biology, Academia Sinica, Taipei, Taiwan; 4 Department of Nephrology, Kidney Research Center, Chang Gung Memorial Hospital, Chang Gung University College of Medicine, Taoyuan, Taiwan; Monash University, Australia

## Abstract

Leptospirosis is one of the most widespread zoonotic diseases in the world. It is caused by the pathogen *Leptospira* that results in multiple-organ failure, in particular of the kidney. Outer membrane lipoprotein is the suspected virulence factor of *Leptospira*. In *Leptospira* spp LipL41 is one major lipoprotein and is highly conserved. Previous study suggests that LipL41 bears hemin-binding ability and might play a possible role in iron regulation and storage. However, the characterization of hemin-binding ability of LipL41 is still unclear. Here the hemin-binding ability of LipL41 was examined, yielding a *K*_*d*_ = 0.59 ± 0.14 μM. Two possible heme regulatory motifs (HRMs), C[P/S], were found in LipL41 at ^140^Cys-Ser and ^220^Cys-Pro. The mutation study indicates that Cys140 and Cys220 might be cooperatively involved in hemin binding. A supramolecular assembly of LipL41 was determined by transmission electron microscopy. The LipL41 oligomer consists of 36 molecules and folds as a double-layered particle. At the C-terminus of LipL41, there are two tetratricopeptide repeats (TPRs), which might be involved in the protein-protein interaction of the supramolecular assembly.

## Introduction

Leptospirosis is one of the most widespread zoonotic diseases in the world and is caused by the pathogen *Leptospira* [1,2]. It is also known as Weil’s syndrome, and the clinical manifestations of leptospirosis occur when humans acquire the pathogen *Leptospira* from animals [3] via skin or gastrointestinal contact with water, food, or soil. Clinical symptoms of leptospirosis include high fever, bleeding, and renal failure [4]. The major target of *Leptospira* in the kidney is the renal proximal tubular cells, and the pretreatment with *Leptospira* outer membrane proteins (OMPs) leads to tubulointerstitial nephritis and acute renal malfunction [2,5,6].

The proteins (OMPs) and lipopolysaccharides on leptospiral outer membrane are the major antigens that result in immunity to *Leptospira* and might be responsible for renal dysfunction [7-9]. Leptospiral OMPs are likely to be involved in the host-pathogen interactions [8,10,11]. They elicit inflammation and lead to tubular injuries through Toll-like receptor-dependent pathways. Subsequently, the nuclear transcription factor NF-κB and the mitogen-activated protein kinases are induced, leading to the differential induction of chemokines and cytokines relevant to tubular inflammation [12-14]. Among three types of outer membrane proteins (transmembrane protein, peripheral membrane protein and lipoprotein) [15], lipoproteins have been identified in many different species of bacteria [16,17]. The precursors of lipoprotein contain a consensus lipobox located at -3 to +1 positions with “[LVI][ASTVI][GAS]C”, which represents the cleavage region of lipoprotein [17]. The cysteine of the lipobox was modified with an *N*-acyl *S*-diacylglycerol group. The modification anchors the lipoproteins to the cell membrane where they function as structural proteins (e.g., murein lipoprotein) or catalytic proteins (membrane-bound enzymes or transport proteins) [16,18]. Many leptospiral lipoproteins have been identified as virulence factors involved in etiology and pathogenesis of leptospirosis.

Iron acquisition is important in pathogenesis [19,20], and iron is an essential cofactor for many enzymes found in nearly all living organisms [21]. The most abundant form of iron in vertebrates is bound within a porphyrin ring named heme [22]. Bacteria can access this compound and utilize the heme iron. Some bacterial pathogens generate TonB-dependent outer membrane receptors that bind hemin, such as *Vibrio cholera* [23], enterohemorrhagic Escherichia coli O157:H7 [24] and *Shigella dysenteriae* [25]. The bound hemin is subsequently internalized with the help of ATP-binding cassette (ABC) transporters. In another heme uptake system, pathogens secrete heme-binding proteins called hemophores that bind heme and transport it to the cell surface to be internalized by specific cell surface receptors. Such a system was identified in *Serratia marcescens* [26] and *Pseudomonas aeruginosa* [27]. In both systems, either hemin or the iron alone can be internalized after released from hemin at the cell surface [28].

LipL41 is the major outer membrane lipoprotein and was first identified on the surface of *Leptospira interrogans* [29]. LipL41 expression is highly conserved among pathogenic *Leptospira* species [30] and is an antigen used as a serodiagnosis target [31,32]. The function of LipL41 is not clear. LipL41 does not induce inflammation [13] and is not essential for acute leptospirosis [33] either. LipL41 has been recognized as a hemin-binding protein [34]. However, the hemin-binding mechanism of LipL41 is still unclear. In this study, we found that LipL41 forms a supermolecule to bind hemin and a hemin-binding pocket composed of two heme regulatory motifs, ^140^Cys-Ser and ^220^Cys-Pro, was identified.

## Materials and Methods

### Expression and purification of LipL41

The *lipL41* gene minus the signal sequence (first 20 amino acids) from *Leptospira santarosai* serovar Shermani was constructed to a plasmid pRSET (Invitrogen), which denoted pRSET-LipL41. Recombinant LipL41 was overexpressed in *E. coli* strain BL21(*DE3*) and induced with 0.5 mM IPTG at 20 °C for 16 hours. The bacterial pellet was obtained by centrifugation (4,000 ×g) and resuspended in lysis buffer (50 mM Tris-HCl/pH 7.9, 500 mM NaCl, and 20 mM imidazole). The resuspended cells were disrupted by sonication. The cell lysate was centrifuged at 18,000 ×g at 4 °C for 20 min. The supernatant was collected and recombinant LipL41 was purified by a Ni-NTA affinity column (GE Healthcare). Then the purified LipL41 was dialyzed against a sample buffer (100 mM Na/K phosphate/pH 6.0 and 200 mM NaCl) for further analysis and assay. The LipL41 mutants, C140A, C220A, and C140A/C220A were generated using the QuikChange Site-Directed Mutagenesis kit (Stratagene). LipL41-C100 was subcloned from pRSET-LipL41 to pET28a through *Nde*I and *Xho*I cutting sites. The expression and purification of mutants was similar to that of the wildtype.

### SDS-PAGE and native-PAGE

The purified proteins were diluted 2-fold in SDS-PAGE sample buffer consisting of 125 mM Tris-HCl (pH 6.8), 14.4 mM β-mercaptoenthaol, 4% sodium dodecyl sulfate (SDS), and 0.1% bromophenol blue in 20% glycerol. After 5 min boiled at 100 °C, the protein samples were analyzed by 15% SDS-polyacrylamide gel electrophoresis (SDS-PAGE) [35] and followed by Coomassie Brilliant Blue staining. For the examination of oligomer formation, the proteins were analyzed by native-PAGE [36]. Briefly, the purified proteins were diluted 2-fold in loading buffer (125 mM Tris-HCl/pH 6.8, 0.1% bromophenol blue, and 20% glycerol). Then the protein samples were conducted in 8% polyacrylamide gel with 375 mM Tris (pH 8.8) at 160 volts and followed by Coomassie Brilliant Blue staining.

### Size exclusion chromatography

Size exclusion chromatography was performed using a Superdex 200 10/300 GL column (GE Healthcare) pre-equilibrated with sample buffer (100 mM Na/K phosphate/pH 6.0 and 200 mM NaCl). The column was calibrated with ribonuclease A (13.7 kDa), carbonic anhydrase (29 kDa), conalbumin (75 kDa), ovalbumin (440 kDa) and thyroglobulin (669 kDa). Protein sample (0.5 mg/ml) was injected into the column through a 0.5 ml loop. Filtration was carried out at a flow rate of 0.5 ml min^-1^. The eluted protein was detected by measuring the absorbance at 280 nm.

### Analytical ultracentrifuge sedimentation velocity

Analytical ultracentrifugation experiments were performed by an analytical ultracentrifuge (Beckman Optima XL-A) equipped with absorbance optics and a Ti-60a titanium rotor. Sedimentation velocity experiments were fulfilled at 15000 rpm at 20 °C in the double sector Epon centerpieces. Multiple scans at different time points were fit to a continuous size distribution by using SEDFIT [37] to determine the sedimentation coefficient and molecular weight.

### Dynamic light scattering

Dynamic light scattering (DLS) was performed at 25 °C with a DynaPro Molecular Sizing Instrument (DynaPro-MS/X, Wyatt Technology). Purified LipL41 (0.1 mg/ml) in 100 mM Na/K phosphate (pH 6.0) and 200 mM NaCl was centrifuged for 10 min at 16,000 ×g followed by injection into a quartz cuvette illuminated by a laser. At least 20 measurements were taken at each measurement. The hydrodynamic radius (R_H_) and molecular weight of LipL41 were calculated assuming a globular conformation model using Dynamics V6 Software (Wyatt Technology).

### Hemin-agarose binding assay

Binding to hemin-agarose was performed essentially as described by Lee [38]. Briefly, 100 μl of hemin-agarose (Sigma-Aldrich) was washed three times in 1 ml of 100 mM Na/K phosphate (pH 6.0) and 200 mM NaCl, and centrifuged at 750 ×g for 5 min. Hemin-agarose was incubated with 200 μl of 0.1 mg/ml purified LipL41 at 37 °C for 1 hour with gentle mixing. After three washes to remove unbound proteins, the hemin-agarose beads were incubated with SDS-PAGE sample buffer boiled at 100 °C for 5 min followed by centrifugation, and the supernatant was subjected to SDS-PAGE analysis. Sepharose 4B (Sigma-Aldrich), agarose bead without hemin, was used as the non-specific binding control. The binding assay was repeated three times independently. For the densitometry analysis of the binding assay, the protein bands from three independent experiments were quantified by ImageJ software [39]. The statistical analyses were performed with Student's *t*-test. Statistically significant were considered as *p*-values of ≤ 0.05.

### Hemin-dependent peroxidase activity

For the assay of peroxidase activity of the hemin bound by LipL41, a microtiter plate (Nunc-Immuno Plate MaxiSorp surface) was coated with LipL41 at various protein amounts (0-8 μg) in protein buffer (100 mM Na/K phosphate/pH 6.0 and 200 mM NaCl) and incubated at 37 °C for 2 hours. The wells were washed with buffer three times to remove uncoated protein. The coated plate was incubated with 20 μg hemin in 100 μl at 37 °C for 1 hour. The unbound hemin was removed, wells were washed three times, and 100 μl of the ready-to-use substrate tetramethylbenzidine/H_2_O_2_ (Invitrogen) was added. After incubation for 20 min with the substrate, the reaction was stopped with 1 N HCl and the absorbance at 450 nm was determined by iMARK microplate absorbance reader (BIO-RAD). A set of wells that was not coated with LipL41 protein (0 μg) was set as blank to exclude the non-specific binding of hemin. A negative control, lysozyme that does not bind to hemin, was included in the assay. The data were fit to the equation *Y* = *B*max × *X*/*(K*_*d*_ + *X*) using GraphPad Prism 5.0 software. *B*max refers to the maximum specific binding in the same units as *Y*, and *K*_*d*_ is the dissociation constant in the same units as *X*. The hemin-binding ability of LipL41 mutants was determined by the same method. Five micrograms of LipL41 was coated on a microtiter plate, and then incubated with 20 μg hemin. The bound hemin was detected following the addition of tetramethylbenzidine by reading the absorbance at 450 nm. The binding ability of LipL41 was represented by the Abs_450_ and normalized to 100%. The relative bindings of mutants were compared and normalized to the wildtype as described by Valcu [40]. The data were expressed as the means ± standard deviation. The statistical analyses were performed with Student's *t*-test. Statistically significant were considered as *p*-values of ≤ 0.05.

### Transmission electron microscope imaging

A 4 μl aliquot of 0.1 mg/ml LipL41 sample was spotted onto a glow-discharged copper grid (200 mesh, Formvar /Carbon 01800-F, Pelco) and incubated for 1 min. Excess buffer was then carefully blotted away from the edge of the grid with filter paper (Whatman Inc., USA). Finally, the grid was stained with 2% uranyl acetate for 40 sec, then the solution was wicked off, and the grid was air-dried. Samples were examined under a transmission electron microscopy (TEM) (Tecnai G2 Spirit TWIN, FEI Company). Electron micrographs were routinely recorded at 21,000X and 52,000X instrumental magnification. The image processing was performed with the EMAN2 software package [41].

## Results

### LipL41

**Figure 1:**
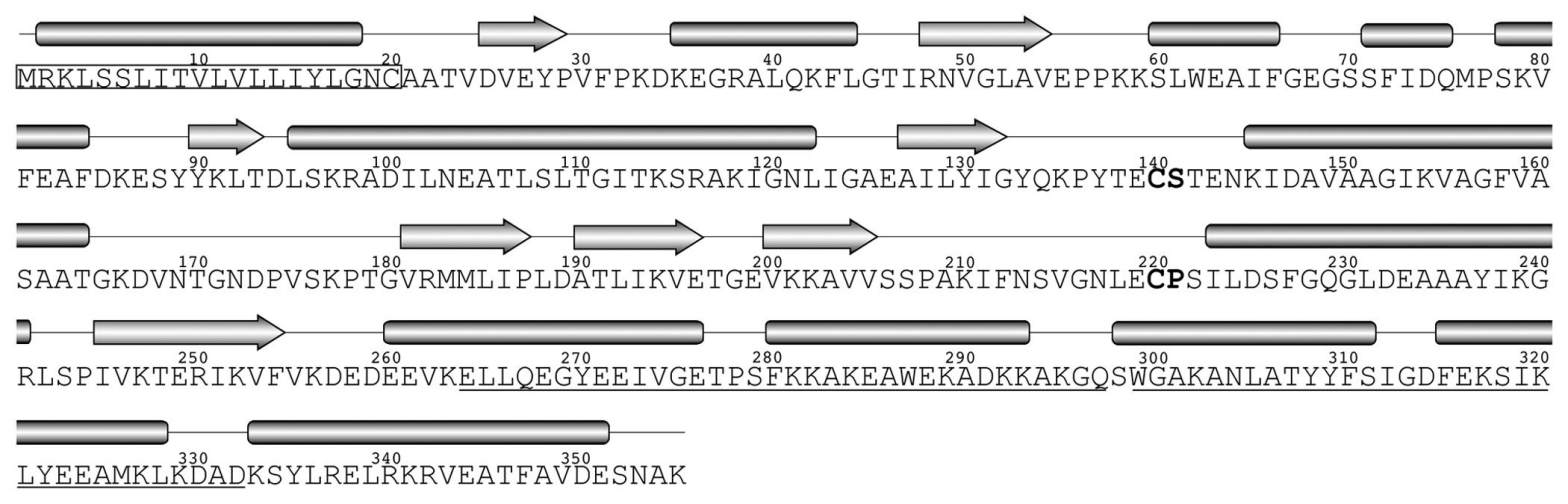
LipL41 sequence and predicted secondary structure. The amino acid sequence and predicted secondary structural elements of LipL41 are shown. The α-helices and β-strands are represented by cylinders and arrows, respectively. The signal peptide for lipidation is boxed. Two putative heme regulatory motifs (HRMs) are shown in boldface. Two putative TPR (tetratricopeptide repeat) motifs are underlined.

LipL41 is highly conserved among *Leptospira* spp. The amino acid sequence and secondary structure prediction of LipL41 are shown in Figure 1. The signal peptide of LipL41, “^1^MRKLSSLITVLVLLIYLGNC^20^”, conforms to the definition of a prokaryotic lipoprotein [17], including a basic region (^1^MRK^3^), a middle hydrophobic region (^4^LSSLITVLVLLIY^16^), and a lipobox (^17^LGNC^20^). No conserved domains of LipL41 were found in the Conserved Domain Database [42]. In the C-terminus, residues 264-297 and 299-332 were predicted as two TPR (tetratricopeptide repeat) motifs by TPRpred [43]. TPR motifs are found in numerous proteins, serving as protein-protein interaction modules and multiprotein complex mediators [44,45]. It is possible that LipL41 utilizes TPR motifs to carry out the protein-protein interaction and form a homo-oligomer. Most of LipL41 is hydrophobic except the C-terminal fragment (residues 256-355), which is a hydrophilic region (Figure S1). This hydrophilic C-terminus may contribute to the solubility of LipL41.

The heme regulatory motif (HRM) has been determined in functionally diverse proteins by simply binding to heme (Fe^2+^)/hemin (Fe^3+^) [46-48]. Heme is a prosthetic group with an iron ion in the center of a large heterocyclic organic ring called porphyrin [22]. It has been reported that the cysteine containing dipeptide, Cys-Pro or Cys-Ser, is necessary for heme binding in HRM [49-51]. These conserved residues are found in LipL41 as ^140^Cys-Ser and ^220^Cys-Pro that are located in the predicted flexible loops (Figure 1), and the thiol of cysteine may play a ligand of iron on heme [51,52]. 

### Supramolecular assembly

The recombinant LipL41 (Ala21 - Lys355) with an N-terminal His_6_-tag was initially expressed at 37 °C and induced by 1 mM IPTG for 3 hours. The overexpressed LipL41 was abundant; however large amounts remained in the inclusion body. This phenomenon is similar to a recent report by King et al. [33], where LipL41 was found to be largely insoluble from the *E. coli* expression system, suggesting that the stable expression of LipL41 needs a chaperone, Lep. In order to obtain the soluble recombinant LipL41, we used the expression vector, pRSET, reduced the concentration of IPTG from 1 to 0.5 mM, decreased the induction temperature from 37 to 20 °C, and prolonged the induction time from 3 to 16 hours. The solubility of LipL41 was increased, and approximately 50% of the total expressed protein was obtained.

**Figure 2:**
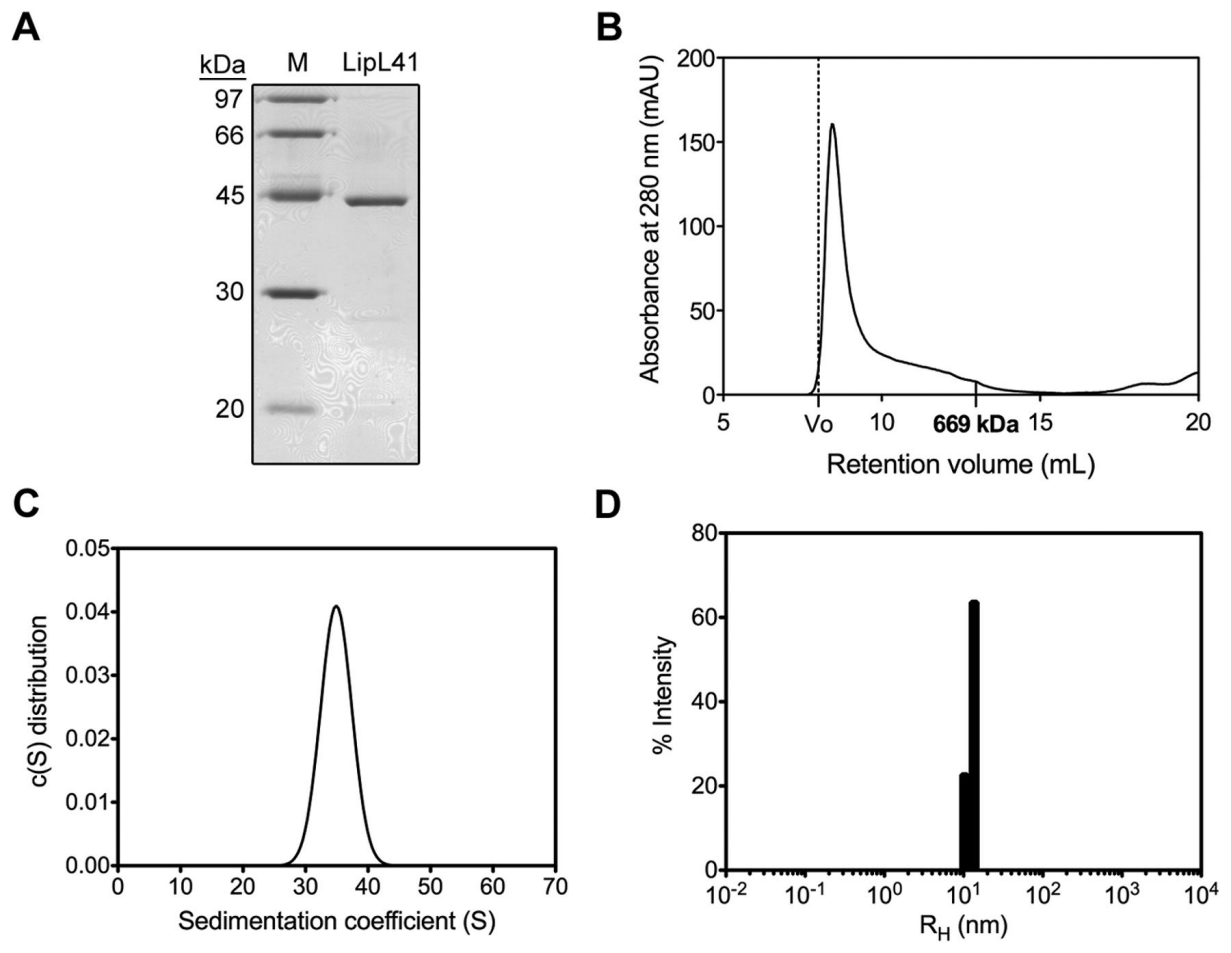
Oligomerization of LipL41. (A) SDS-PAGE (15%) analysis of LipL41. The molecular weight markers (M) are shown. (B) Size-exclusion chromatography of LipL41. LipL41 was analyzed by Superdex 200 and a single peak after the void volume (dot line) was observed. The retention volume of a standard protein (thyroglobulin, 669 kDa) is indicated. (C) The sedimentation coefficient of LipL41 by analytical ultracentrifugation. The sedimentation coefficient of LipL41 was determined to be a value of 35S. (D) Dynamic light scattering analysis of LipL41. LipL41 was shown to be monodisperse with a polydispersity (%Pd) of 11.5%. The equivalent hydrodynamic radius (R_H_) was 12.7 nm. The estimated molecular weight was calculated to be 1296 kDa.

LipL41 was examined by SDS-PAGE, revealing an estimated molecular weight of 40 kDa (theoretical MW = 40.1 kDa) (Figure 2A). However, the LipL41 sample was lodged in the 8% native-PAGE loading well (data not shown). This result indicates that LipL41 might have formed a large oligomer or even aggregated in the sample. To further investigate the oligomerization or aggregation of LipL41, size-exclusion chromatography and analytical ultracentrifugation were employed. The molecular weight of LipL41 oligomer was determined to be higher than the 669 kDa marker protein, thyroglobulin, in size-exclusion chromatography (Superdex 200) (Figure 2B). Also, the sedimentation coefficient of LipL41 was determined by analytical ultracentrifugation as 35S (Figure 2C), and the molecular weight of LipL41 was estimated to be around 1000 kDa.

Furthermore, the homogeneity of LipL41 sample was determined by dynamic light scattering (DLS). The hydrodynamic radius (R_H_) distribution appeared as a single peak (Figure 2D) and the polydispersity was measured as 11.5%, indicating that LipL41 exists as a single conformation, not an aggregation in solution. The equivalent hydrodynamic radius was calculated to be 12.7 nm, corresponding to an estimated molecular weight of 1296 kDa that is similar to that from analytical ultracentrifugation (Figure 2C). These results demonstrated that the LipL41 forms a stable supermolecule in solution.

The supramolecular assembly of LipL41 may be caused by the C-terminal TPR-containing segment. To examine whether the TPR motifs lead to LipL41 oligomer formation, we made two recombinant proteins, the C-terminal-truncated LipL41 (residues 21-255, LipL41ΔTPR) and LipL41-C100 with a TPR-containing fragment of residues 256-355. Unfortunately, the LipL41ΔTPR was insoluble and formed an inclusion body during expression (data not shown). Nevertheless, LipL41-C100 was analyzed by size-exclusion chromatography and analytical ultracentrifugation to form a dimer in the solution (data not shown). These results suggest that the TPR motifs in the C-terminus might promote the oligomer formation of LipL41. Meanwhile, the hydrophilicity of the C-terminus of LipL41 (LipL41-C100, Figure S1) might result in improved solubility of LipL41.

### TEM analysis

**Figure 3:**
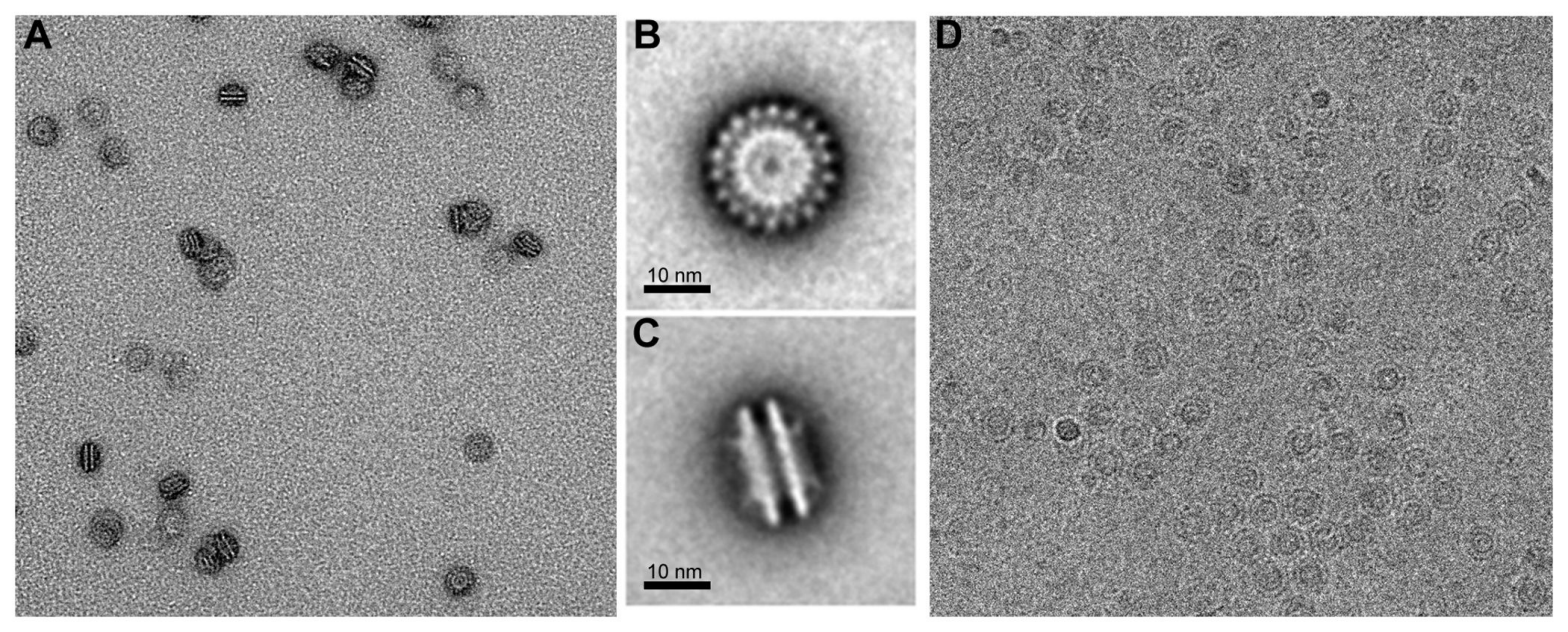
Electron micrographs of LipL41. (A) TEM image of LipL41. The LipL41 with uranyl acetate negative staining existed as uniform and homogeneous particles. Class averages of LipL41 supermolecules are shown in top (B) and side views (C) with the scale bar 10 nm. The repeat of eighteen molecules was estimated from the top view of the particle (B), and each particle forms a two-layered structure from the side view (C). (D) Cryo-EM field of ice-embedded particles of LipL41. The shape of the particles is identical to LipL41 with negative staining (A), showing that negative staining did not interfere with particle formation.

Transmission electron microscope (TEM) was applied to visualize the oligomeric LipL41 in solution (Figure 3). The electron micrograph shows that LipL41 exists as a uniform and homogeneous particle (Figure 3A). The two representative class averages of LipL41 particles were computed and a double-layered yo-yo shape oligomer was determined (Figure 3B and C). The LipL41 particle was calculated with a 23 nm × 13 nm dimension that coincides with the hydrodynamic radius obtained from DLS analysis of 12.7 nm (Figure 2D). A repeat of eighteen molecules was observed in the top view of LipL41 particle (Figure 3B) and a double-layered structure was apparent (Figure 3C). Therefore, LipL41 forms a large oligomer consisting of 36 units in total (18 units per layer).

Meanwhile, TEM was used to measure the LipL41 particle embedded in vitreous ice (Figure 3D), which is identical to the LipL41 treated with uranyl acetate negative staining (Figure 3A). Results showed that the negative staining does not interfere with the particle conformation. The cryo-EM image (Figure 3D) and uranyl acetate negative staining (Figure 3A) of LipL41 particle are identical, suggesting that LipL41 forms a uniform and homogeneous particle in solutions. In our single particle analysis result (unpublished data), the volume of the 36 subunits of LipL41 particle was computed to be 1.8×10^6^ Å^3^ with a protein density of 1.3 g/cm^3^, which coincides with the average protein density of 1.2-1.4 g/cm^3^ [53].

### Hemin binding ability of LipL41

**Figure 4:**
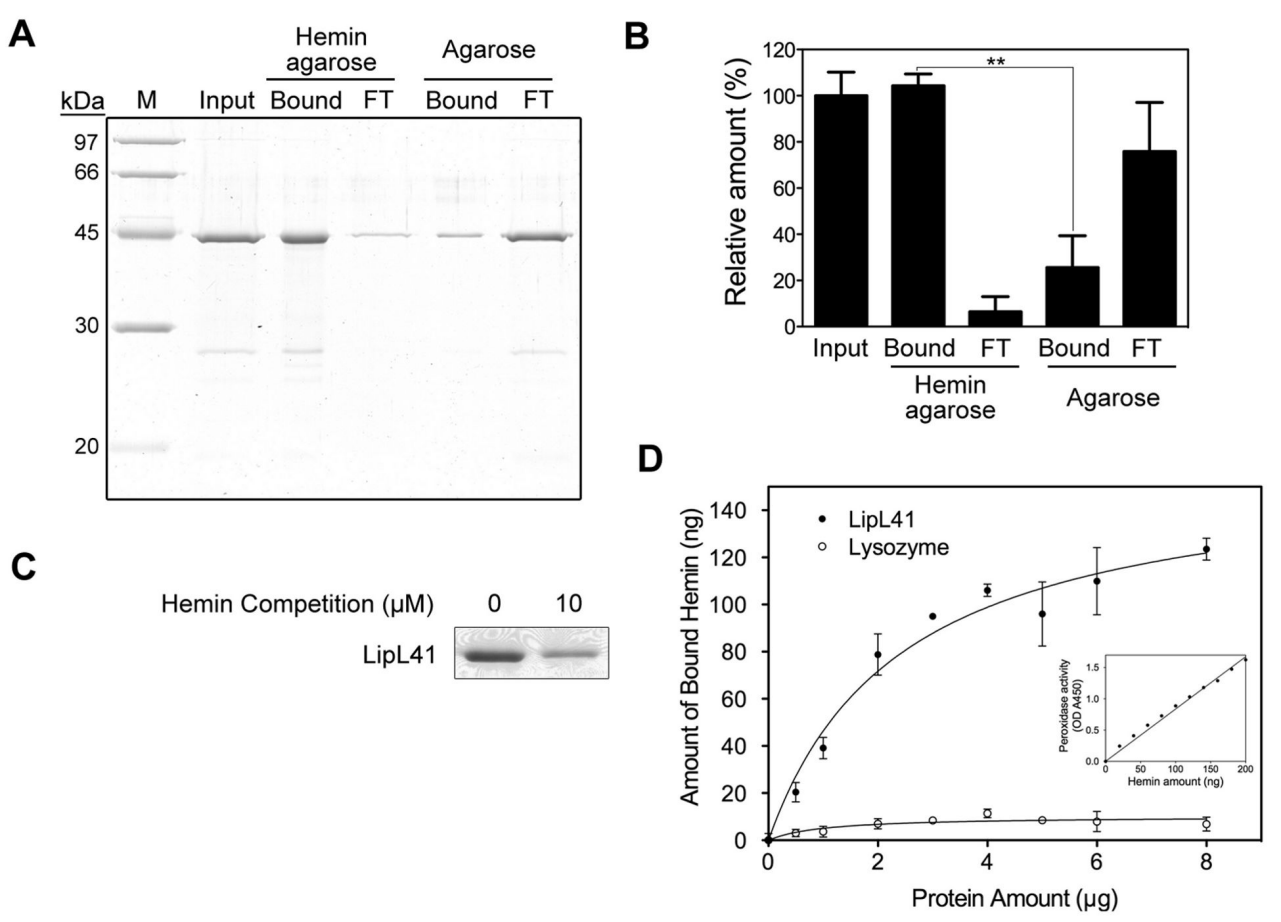
Hemin-binding ability of LipL41. (A) The hemin binding of LipL41 was examined by hemin-agarose beads. Shown are input sample (Input), hemin-agarose-bound (Bound) and resulting flow through (FT), and the non-specific binding control of agarose-bound (Bound) and resulting flow through (FT). Agarose without hemin was the non-specific binding control. The experiments were repeated three times independently. (B) The results from (A) were quantitated by ImageJ software and the difference of binding to hemin-agarose and to agarose alone was determined by Student’s *t*-test (**: *p*-value < 0.01). (C) LipL41 was incubated with 0 or 10 μM hemin prior to binding with hemin-agarose beads. The hemin-binding ability of LipL41 was competed by preincubating with hemin. (D) The amount of hemin bound by LipL41 was estimated by inherent hemin-dependent peroxidase activity. A microtiter plate was coated with various protein amounts (0-8 μg) of LipL41 (solid symbol). Lysozyme was the negative control (open symbol). The peroxidase activity of the bound hemin was assayed by the addition of tetramethylbenzidine and reading of the absorbance at 450 nm. The inset shows the standard graph of peroxidase activity of hemin. The amount of bound hemin was increased until about 3 μg of LipL41, when the hemin reached a plateau level of about 100 ng of hemin with a molar ratio of 0.5 (LipL41 to hemin). The *K*_*d*_ was calculated to be 0.59 ± 0.14 μM. The statistical significance of hemin binding was determined by Student’s *t*-test with a *p*-value of 0.001.

A previous study using hemin-agarose beads proposed that LipL41 has hemin-binding properties [34]; however, King et al. [33] reported that LipL41 from whole cell lysate exhibited non-specific binding to agarose beads while its hemin binding could not be observed. Thus the hemin-binding ability of LipL41 was uncertain. To characterize the hemin-binding ability, we examined the recombinant LipL41 by hemin-conjugated agarose and found that a substantial amount of LipL41 was captured by hemin-agarose resin. Although some non-specific binding of LipL41 to agarose beads was found, the binding to hemin-agarose was much stronger than to agarose without hemin (Figure 4A and B). Further, competition was observed following prior incubation with free hemin (Figure 4C). These results suggest that LipL41 has a specific binding affinity to hemin.

In addition, the hemin-binding ability of LipL41 was quantified by the heme-dependent peroxidase activity assay (Figure 4D). Various amounts of LipL41 protein (0-8 μg) were coated on a microtiter plate, and the amount of bound hemin increased dose-dependently with coated protein. At 3 μg of LipL41, the binding reached a saturation of about 100 ng hemin with a molar ratio of 0.5 (LipL41 to hemin). This indicated that one LipL41 binds two hemin molecules. The dissociation constant (*K*_*d*_) of hemin binding to LipL41 was calculated to be 0.59 ± 0.14 μM, which is similar to that of other hemin-binding proteins in a sub-micromolar range [52,54].

**Figure 5:**
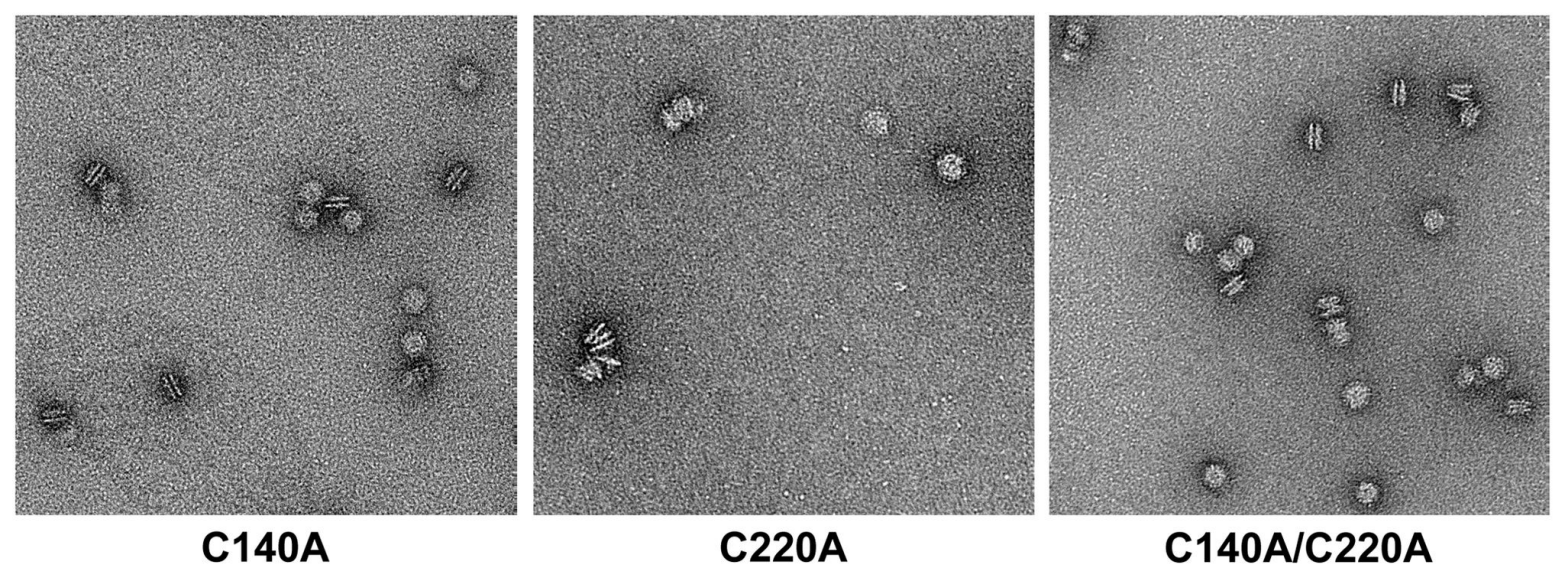
Electron micrographs of LipL41 HRM mutants. TEM images of LipL41 HRM mutants with negative staining are shown. Mutations of Cys140 or/and Cys220 to alanine had no effect on the particle formation.

**Figure 6:**
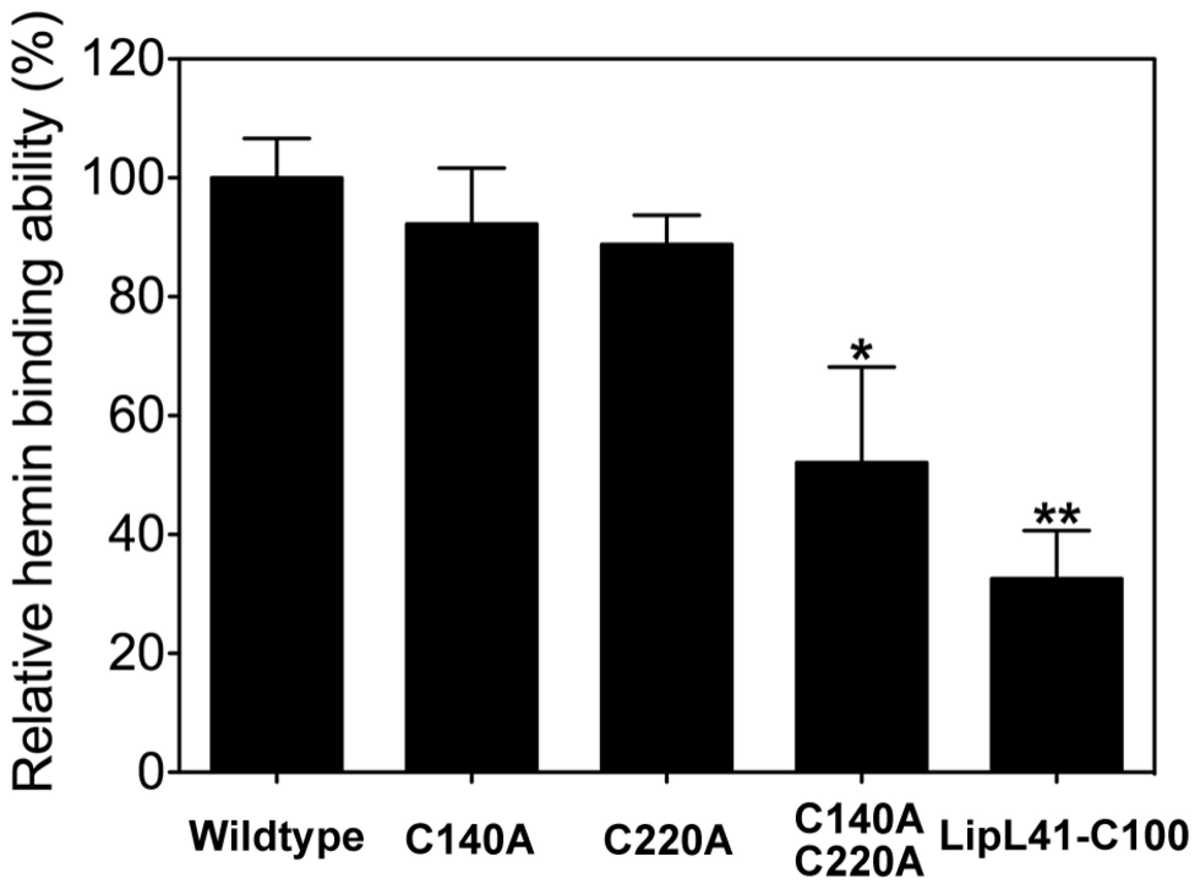
Hemin-binding ability of LipL41 mutants. The relative hemin-binding ability of LipL41 wildtype, C-terminal region of LipL41 (LipL41-C100), and three cysteine mutants (C140A, C220A and C140A/C220A) were shown by hemin dependent peroxidase activity. The cysteine point mutations, C140A and C220A, decreased the binding ability slightly to 92% and 89%, respectively. The cysteine double mutant, C140A/C220A, retained 52% of hemin-binding ability. The LipL41-C100 retained only 33% of binding ability. Results are representative of three independent experiments. The significant difference between wildtype and mutants was determined by Student’s *t*-test. *: *p*-value < 0.05, **: *p*-value < 0.01.

The heme regulatory motif (HRM) may participate in hemin binding, and two possible HRMs were identified ^140^,Cys-Ser and ^220^Cys-Pro (Figure 1). From the disulfide bond prediction determined by the EDBCP programs [55], Cys140 and Cys220 do not form a disulfide bond. These two free cysteines in LipL41 might be involved in the hemin binding. To localize the hemin-binding motif, three HRM mutations, C140A, C220A, and C140A/C220A, were constructed. TEM examination revealed that the supermolecule formations of these HRM-mutated LipL41 proteins were not affected (Figure 5). We further evaluated the hemin-binding ability of these mutants by hemin-dependent peroxidase activity. By comparing to the wildtype LipL41, the binding ability of C140A and C220A decreased slightly; however, that of double mutant C140A/C220A was significantly decreased to 52% (Figure 6). It is possible that Cys140 and Cys220 interact with hemin cooperatively. Despite the fact that mutation of both cysteines decreased the hemin-binding ability significantly, it still retained 52% binding ability. This suggests that there are other residues corresponding to hemin binding. Further, LipL41-C100 retained only 33% of the wildtype hemin binding, suggesting that the N-terminal region (residues 21-255) of LipL41 contributes considerably to the hemin-binding ability.

## Discussion

Iron is an essential factor in growth and virulence for most pathogenic microbes, including *Leptospira* spp [56]. To cope with iron acquisition, pathogens have evolved diverse mechanisms for obtaining iron molecules. In a mammalian host, the availability of free iron is highly restricted with heme being the most abundant form of organic iron; therefore the ability to utilize heme compounds is important in pathogenic bacteria. However, the mechanisms of iron acquisition and regulation are still unclear in *Leptospira*. Recently, several outer membrane proteins have been reported to be iron regulated in pathogenic *Leptospira* [57]. For example, HbpA is up regulated [34] and LipL36, pL24, and pL50 are down regulated [58] under low iron conditions. In this study, we characterized the hemin-binding ability of LipL41. LipL41 is highly conserved among pathogenic *Leptospira* spp, and expressed constitutively without the regulation of iron level [34]. Asuthkar et al. indicated that LipL41 was bound by hemin-agarose in outer membrane of several leptospiral species, even in the nonpathogenic *L. biflexa* [34]; however, the orthologous genes of some characterized lipoprotein, such as LipL41, LipL32, LipL36 and several LipL45 related proteins, were not encoded in *L. biflexa* [59,60]. We determined the hemin-binding ability of LipL41 from *L. santarosai* serovar Shermani by hemin-agarose binding assay (Figure 4A, B and C), revealing that LipL41 binds hemin specifically. The amount of hemin bound by LipL41 and the dissociation constant (*K*_*d*_) were estimated by heme-dependent peroxidase activity (Figure 4D). The *K*_*d*_ was calculated to be 0.59 ± 0.14 μM, and the binding affinity of LipL41 in the sub-micromolar range was similar to other hemin-binding proteins. The stoichiometry of hemin to LipL41 is estimated to be two, that is to say, one LipL41 can bind two hemin molecules. LipL41 was suggested to possess two heme regulatory motifs (HRMs) ^140^,Cys-Ser and ^220^Cys-Pro. When we mutated these two cysteines of HRM, the hemin-binding ability decreased significantly (Figure 6). However, single mutation on either Cys140 or Cys220 alone did not impair the binding ability. This implies that these two HRMs might form a hemin-binding pocket, and the cysteines coordinate with hemin cooperatively. Since 52% of binding ability is retained, there are likely additional binding pockets that have not been identified. Since the hemin-binding ability of LipL41-C100 was not observed, the hemin-binding motifs might be located at the N-terminal region of LipL41.

Recently, King et al. suggests that LipL41 does not have hemin-binding ability by hemin-agarose assay and spectral analysis [33]. They used cell lysate of *L. interrogans* to perform hemin-agarose binding assay and revealed that LipL41 bound agarose in the absence of hemin. Instead of using cell lysate, we used purified recombinant protein to perform the similar assay and found that LipL41 has a specific binding affinity to hemin. The diverse phenomenon observed by King et al. maybe due to the effects from other proteins in the whole cell lysate.

Organisms have evolved proteins capable of reversibly storing iron, and many of these iron-storing proteins form a high order oligomer [61]. Ferritins in mammals and bacteria are all composed of 24 subunits assembled to form a spherical protein. The frataxin is an oligomer of 48 subunits. Of particular note, the *E. coli* bacterioferritin (*Ec*BFR), which is an iron storage and detoxification protein, forms a 24mer with 12 hemes bound [61,62]. In this study, we found that a supermolecule LipL41 made up of 36 subunits arranged in a yo-yo particle reveals the hemin-binding ability. The features of supramolecular assembly and hemin-binding ability give LipL41 a high potential for being an iron-storing protein.

## Supporting Information

Figure S1 **LipL41 hydrophobicity plot.** The Kyte-Doolittle hydrophobicity plot of LipL41 shows that the signal peptide is the most hydrophobic region and the C-terminal region (amino acids 250 through 355) is hydrophilic significantly. The amino-terminal and middle region are moderate hydrophobic.
(TIF)
