# Supplementary figures and images for: LipL41, a Hemin Binding Protein from Leptospira santarosai serovar Shermani
Source: PLoS One. 2013 Dec 12;8(12):e83246. doi: 10.1371/journal.pone.0083246 (PMC3861479; doi:10.1371/journal.pone.0083246)

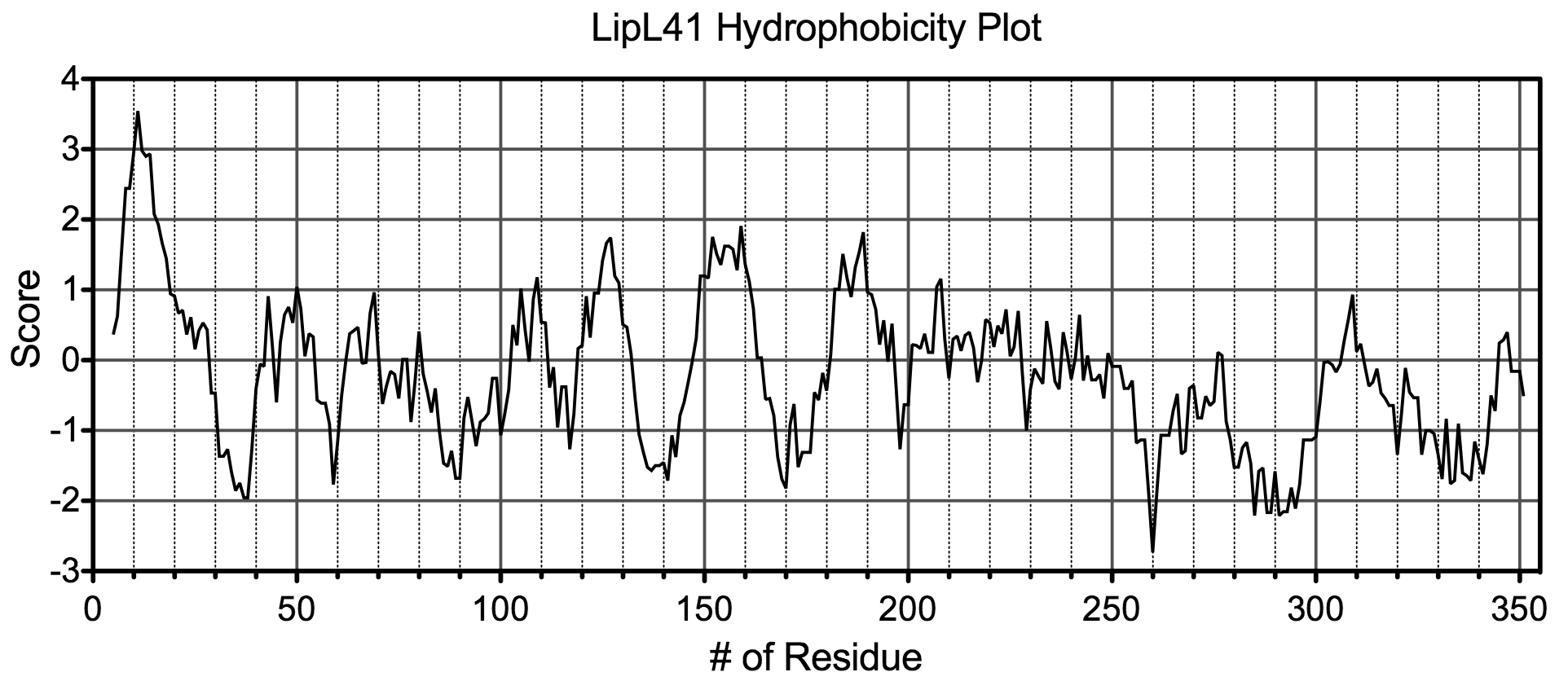

Supplement: Figure S1 — LipL41 hydrophobicity plot. The Kyte-Doolittle hydrophobicity plot of LipL41 shows that the signal peptide is the most hydrophobic region and the C-terminal region (amino acids 250 through 355) is hydrophilic significantly. The amino-terminal and middle region are moderate hydrophobic. (TIF) [file pone.0083246.s001.tif]
